# Beyond the Surface: A Clinical Insight Into a 60-Year-Old Male With Pemphigus vulgaris

**DOI:** 10.7759/cureus.54408

**Published:** 2024-02-18

**Authors:** Miis Akel, Maggie Wright, Bialo Aladum, Sergio Hernandez Borges

**Affiliations:** 1 Medicine, Dr. Kiran C. Patel College of Osteopathic Medicine, Nova Southeastern University, Clearwater, USA; 2 Dermatology, Dr. Kiran C. Patel College of Osteopathic Medicine, Nova Southeastern University, Miami, USA; 3 Psychiatry, Ascension Borgess Hospital, Kalamazoo, USA; 4 Internal Medicine, Family Medicine, Larkin Community Hospital Palm Springs Campus, Hialeah, USA

**Keywords:** pemphigus group, oral mucosal lesions, acantholysis, clinical dermatology, pemphigus vulgaris

## Abstract

Pemphigus vulgaris, a rare and life-threatening autoimmune disorder, presents with painful skin and mucosal lesions, leading to blistering sores attributed to acantholysis. This study delves into the clinical manifestations, risk factors, and diagnostic intricacies associated with pemphigus vulgaris, with a focus on a representative case highlighting the challenges in its recognition and management. We explore the case of a 60-year-old male with pemphigus vulgaris, whose initial presentation involved yellow-crusting lesions on the scalp progressing to non-pruritic lesions on the chest, neck, and inguinal areas. A multidisciplinary medical workup was conducted, encompassing serological tests, imaging, and consultations with infectious disease and dermatology specialists. The definitive diagnosis was established through histopathological examination of three 4-mm punch biopsies. The case underscores the polymorphic nature of pemphigus vulgaris, with diverse clinical presentations and diagnostic challenges. The positive Nikolsky sign on the chest and neck lesions, coupled with oral mucosal involvement observed during a routine dental procedure, exemplifies the complexity of its manifestations. Diagnostic intricacies involved negative results for infectious diseases, declined kidney function, and elevated inflammatory markers, necessitating a collaborative approach for accurate diagnosis. Pemphigus vulgaris demands a comprehensive understanding of its varied presentations and collaboration among medical specialties for accurate diagnosis and tailored management. Treatment involves systemic glucocorticoids and immunomodulators. The presented case underscores the need for continued research to enhance diagnostic accuracy and refine therapeutic interventions for this rare autoimmune disorder.

## Introduction

Pemphigus vulgaris, a rare autoimmune disorder, manifests through painful skin and mucosal lesions that progress into blistering sores because of acantholysis, defined as the loss of keratinocyte adhesions. Populations at increased risk for pemphigus vulgaris include the Ashkenazi Jews, Indians, Southeast Europeans, and Middle Easterners [1]. Typically afflicting adults between the ages of 40 and 60, this disease exhibits an equal predilection for both genders [1]. Genetic predisposition is apparent, with associations observed between pemphigus vulgaris and HLA class II alleles DR4 and DR14 [2], the specific genes implicated varying according to ethnic backgrounds. Individuals with first-degree relatives affected by pemphigus exhibit an increased prevalence of autoimmune diseases [1], often correlated with autoimmune thyroid disease, rheumatoid arthritis, and type 1 diabetes within affected families.

Drug-induced pemphigus, triggered by drugs such as penicillin, cephalosporins, enalapril, rifampin, and nonsteroidal anti-inflammatory drugs (NSAIDs), is believed to induce acantholysis by interfering with enzymes mediating keratinocyte aggregation or binding molecules in cell adhesion.

Distinguishing characteristics set pemphigus apart from pemphigoid disorders, notably bullous pemphigoid. Pemphigus presents with shallow ulcers and fragile blisters, easily breaking open and exhibiting a positive Nikolsky sign, while pemphigoid displays stronger, tense blisters resistant to rupture [3]. Acantholysis in pemphigus results from immunoglobulin G (IgG) autoantibodies binding to intercellular adhesion molecules. Pemphigus vulgaris further differentiates from pemphigus foliaceus by involving mucosal surfaces, featuring IgG autoantibodies against desmoglein 3 or both desmoglein 1 and 3, whereas foliaceus is confined to the skin with autoantibodies against desmoglein 1 [1].

Nearly all pemphigus vulgaris patients develop oral mucosal involvement [2], often marking the initial presentation. Additional affected sites include the nose, esophagus, conjunctiva, vulva, vagina, cervix, and anus. Oral mucosa involvement, exacerbated by chewing and swallowing, can lead to unintended weight loss and malnutrition. Cutaneous lesions with easily rupturing blisters, indicating a positive Nikolsky sign, are common, while the palms and soles remain unaffected. Pruritus is typically absent.

Diagnosis requires a 4-mm punch biopsy or detecting IgG autoantibodies against cell surface antigens in serum [1]. Histological findings in pemphigus vulgaris reveal characteristic intraepithelial cleavage with acantholysis localized to the suprabasal region, retention of basal keratinocytes along the basement membrane zone, and sparse inflammatory eosinophilic infiltrates in the dermis.

Given its life-threatening nature, treatment for pemphigus vulgaris is imperative. Objectives encompass achieving complete remission while mitigating treatment-related adverse effects. Long-term remission post-therapy discontinuation is the aim. Initial treatment involves systemic glucocorticoids alongside immunomodulators such as rituximab [4]. Rapid disease control is facilitated by systemic glucocorticoids, with azathioprine and mycophenolate serving as alternatives if rituximab is contraindicated [5]. Once disease control is attained, prednisone can be tapered off. Supportive therapies targeting wound care and alleviating oral symptoms contribute to improved quality of life.

## Case presentation

Presenting complaint

**Figure 1:**
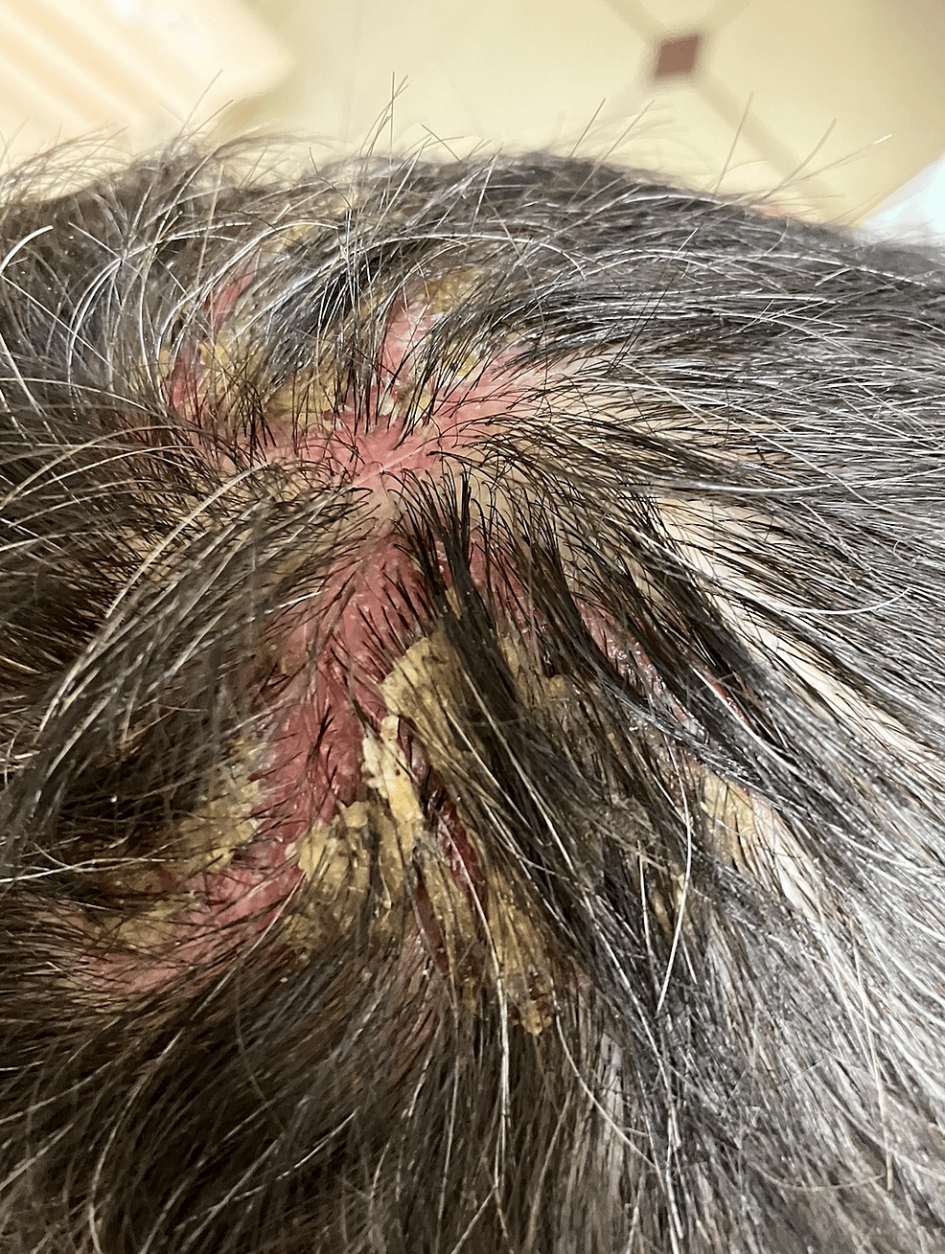
Erythematous scalp and honey-crusted lesions on the scalp

**Figure 2:**
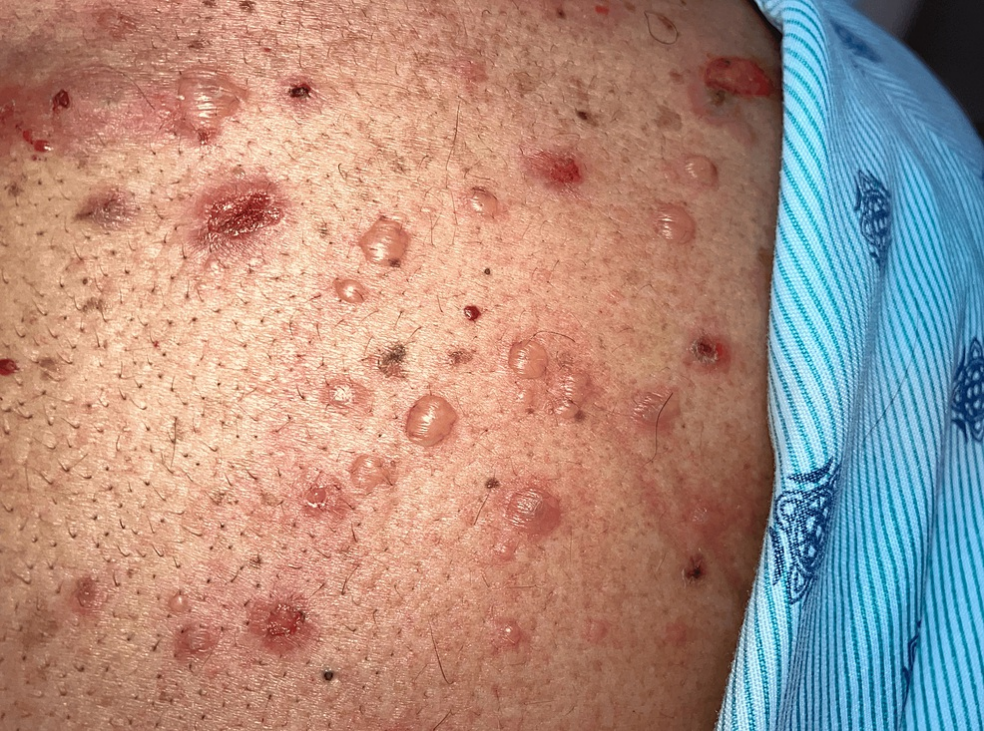
Blisters and sores on the right scapular region

A 60-year-old male with a known medical history of hypertension sought medical attention for the presence of a rash in the emergency department (ED). The patient reported that the skin lesions initially appeared on the scalp a month ago, manifesting as yellow-crusting lesions (Figure 1). He attempted to address the scalp lesions with an unspecified shampoo with no improvement. Similar lesions were noted on the chest, neck, and inguinal areas that were non-pruritic (Figure 2).

Dental discovery

During a routine dental procedure before the patient's ED visit, the patient was observed to have honey-crusted lesions around the nares and angles of the mouth. This prompted the dentist to recommend a dermatologist consultation. Subsequently, the patient presented to the ED and was admitted to the floor by the family medicine department.

Clinical history

The patient reported mild pruritus associated with the scalp lesions (Figure 1), which were otherwise painless and devoid of discharge. In contrast, the lesions on the chest, neck, and inguinal areas were non-pruritic, painful, and lacked discharge (Figure 2). On a physical exam, lesions on the chest and neck had a positive Nikolsky sign. Oral lesions were noted on the physical exam. There were no constitutional symptoms, and the patient denied any previous episodes of similar skin lesions.

Medical workup

**Figure 3:**
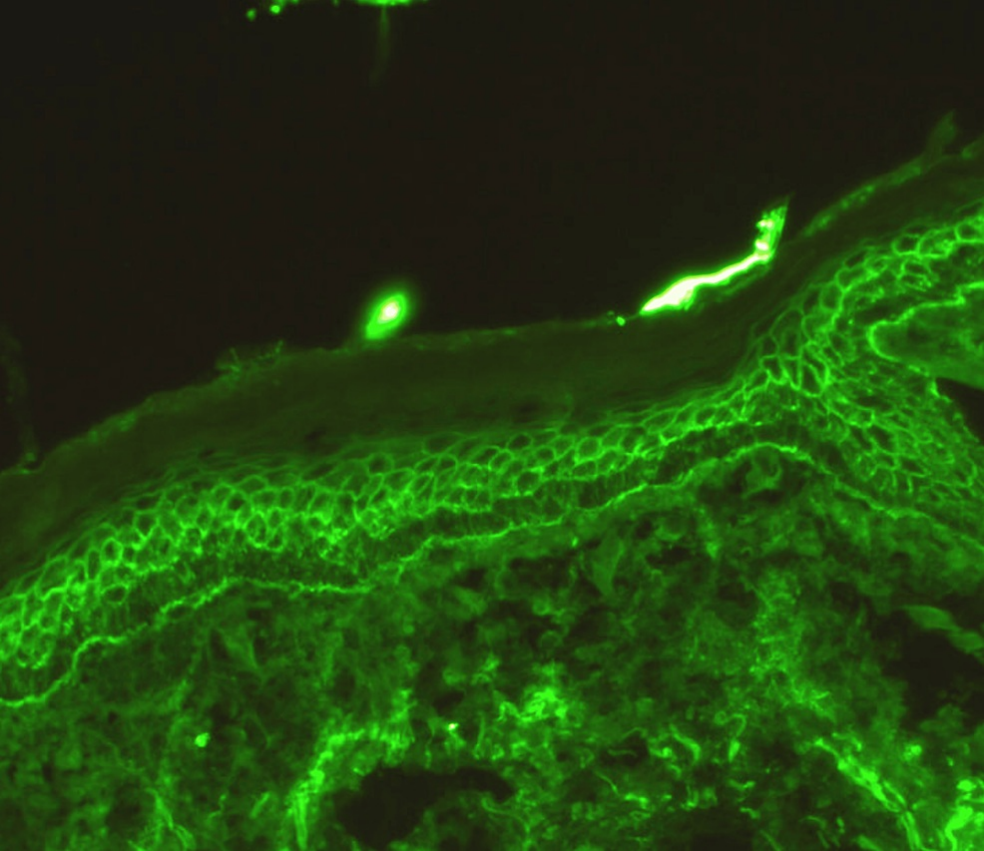
An image showing immunological analysis of the anti-desmoglein 3 antibody Beutner Laboratories. Pemphigus: Laboratory Testing [Internet]. Beutner Laboratories (cited 2024 Feb 13). Available from https://www.beutnerlabs.com/pemphigus-laboratory-testing

The patient underwent a comprehensive medical workup, including a QuantiFERON® (Cellestis Ltd., Melbourne, Australia) gold test, serum protein electrophoresis (SPEP), urine protein electrophoresis (UPEP), and a human immunodeficiency virus (HIV) test, which were all negative. The patient’s wound and blood cultures were negative, along with a nasal culture which ruled out methicillin-resistant staphylococcus aureus (MRSA) infection. A complete blood cell count (CBC) showed a white blood cell (WBC) count of 10,100 per microliter, a red blood cell count (RBC) of 14.5 million per cubic millimeters, and a platelet count of 237,000 per microliter. A complete metabolic panel revealed a sodium level of 138 milliequivalents per liter (mEq/L), potassium of 3.7 mEq/L, chloride of 97 mEq/L, bicarbonate of 32 millimoles per liter (mmol/L), a blood urea nitrogen (BUN) of 23 mmol/L, an elevated creatinine of 1.77 milligrams per deciliter (mg/dL), and blood glucose of 104 mg/dL. His kidney function declined with a glomerular filtration rate of 39 mL/min. His inflammatory markers were elevated with an erythrocyte sedimentation rate (ESR) at 22 mm/hr, and a c-reactive protein high sensitivity (CRPHS) at 14.1 mg/L. A urinalysis was performed with results within normal limits, urine color was light yellow, and composition was negative for glucose, ketones, proteins, nitrites, and leukocyte esterase. His chest X-ray imaging resulted in no focal pulmonary consolidations, pleural effusions, or cardiomegaly, indicating an absence of acute cardiopulmonary disease. Infectious disease and dermatology were both consulted on this case, and their recommendations were considered on this case with wound and blood cultures and skin biopsy. The definitive diagnosis was established through three 4-mm punch biopsies that revealed the presence of rounded-up and separate keratinocytes (acantholytic cells) just above the basal layer of the epidermis. Pathology findings confirmed autoantibodies of IgG against desmoglein 3 (Figure 3). These findings supported a diagnosis of pemphigus vulgaris.

## Discussion

Pemphigus vulgaris, a rare autoimmune disorder characterized by acantholysis and mucocutaneous lesions, poses diagnostic challenges and necessitates a comprehensive understanding of its clinical manifestations, risk factors, and diverse presentations. The presented case underscores the complexity of diagnosing pemphigus vulgaris and highlights the importance of a multidisciplinary approach involving dermatology, infectious disease, and family medicine.

The case of a 60-year-old male with pemphigus vulgaris is emblematic of the varied and often perplexing clinical presentations associated with this autoimmune condition. The initial manifestation on the scalp with yellow-crusting lesions and subsequent progression to non-pruritic lesions on the chest, neck, and inguinal areas emphasizes the polymorphic nature of pemphigus vulgaris. Notably, the positive Nikolsky sign observed on the chest and neck lesions aligns with the classic features of pemphigus, distinguishing it from pemphigoid disorders [3-13]. The patient's oral mucosal involvement, initially identified during a routine dental procedure, further underscores the importance of a thorough clinical examination, especially in cases where skin lesions may not be the primary concern. The absence of constitutional symptoms and the denial of previous similar episodes highlight the insidious onset of pemphigus vulgaris, making early detection challenging.

The diagnostic journey in this case involved an extensive medical workup, including negative results for infectious diseases, SPEP, and UPEP. The patient's declined kidney function and elevated inflammatory markers added complexity to the diagnostic process, necessitating a collaborative effort between infectious disease and dermatology specialists.

The definitive diagnosis of pemphigus vulgaris was achieved through histological examination of three 4-mm punch biopsies, revealing characteristic acantholytic cells just above the basal layer of the epidermis [1,8-10]. This highlights the pivotal role of histopathology in confirming the diagnosis and guiding appropriate therapeutic interventions.

Treatment of pemphigus vulgaris is imperative because of its life-threatening nature [4-6,12,13]. The presented case aligns with established therapeutic goals, emphasizing the use of systemic glucocorticoids in combination with immunomodulators such as rituximab for achieving rapid disease control [5]. The consideration of alternative agents, including azathioprine and mycophenolate, underscores the importance of individualized treatment plans based on patient characteristics and contraindications. The multidisciplinary collaboration between infectious disease and dermatology specialists in this case facilitated a comprehensive approach, ensuring the exclusion of secondary causes and guiding the therapeutic strategy.

## Conclusions

The complexity of pemphigus vulgaris is evident in the presented case, emphasizing the necessity for a holistic diagnostic approach and collaborative management. The recognition of diverse clinical presentations, risk factors, and the significance of histopathological confirmation underscores the challenges in addressing this rare autoimmune disorder. Further research and clinical studies are warranted to enhance our understanding of pemphigus vulgaris, ultimately contributing to improved diagnostic accuracy and tailored therapeutic interventions.
